# High circulating activin A plasma levels are associated with tumour stage and poor survival in treatment-naive lung squamous cell cancer patients

**DOI:** 10.1016/j.tranon.2024.102153

**Published:** 2024-10-15

**Authors:** Katharina Sinn, Ahmed Elbeialy, Berta Mosleh, Clemens Aigner, Karin Schelch, Viktoria Laszlo, Balazs Dome, Mir Alireza Hoda, Michael Grusch

**Affiliations:** a Department of Thoracic Surgery, Medical University of Vienna, Vienna, Austria; b Center for Cancer Research, Medical University of Vienna, Vienna, Austria; c Department of Tumour Biology, National Korányi Institute of Pulmonology, Budapest, Hungary; d Department of Thoracic Surgery, National Institute of Oncology, Semmelweis University, Budapest, Hungary; e Department of Translational Medicine, Lund University, Lund, Sweden

**Keywords:** Lung squamous cell carcinoma, Activin A, Biomarker, Prognostic factor

## Abstract

**Objectives:**

Lung squamous cell carcinoma (LUSC) is associated with a poor prognosis and a lack of specific treatment options. The dysregulation of activin A (ActA) has been reported in various malignancies. Herein, we investigated the diagnostic and prognostic significance of ActA in LUSC.

**Materials and methods:**

ActA concentrations were measured using ELISA in plasma samples of 128 LUSC patients (stage I-IV) and 73 controls, and correlated those values with clinicopathological parameters and survival.

**Results:**

ActA plasma levels were significantly higher in therapy-naive LUSC patients compared to controls (444.1 ± 310.9 pg/mL vs 338.9 ± 145.5 pg/mL, *p* = 0.010). ActA levels significantly correlated with advanced stage as well as with T and N factors. High circulating ActA levels were significantly increased in metastatic disease patients compared to M0 disease. Further, patients with ActA levels above a computationally established optimal cut-off value of 443.0 pg/mL had a significantly worse median overall (OS, 17.63 vs 64.77 months, HR 0.391, 95 % CI 0.200–0.762, *p* < 0.001) and median disease-/progression-free survival (DFS/PFS; 11.57 vs 30.20 months, HR 0.502, 95 % CI 0.248–1.019, *p* = 0.020). Multivariate analysis revealed that high ActA levels were an independent prognostic factor for shorter OS (*p* = 0.001) and DFS/PFS (*p* = 0.018). A newly developed score combining CRP and ActA levels was also an independent prognostic factor for OS and DFS/PFS.

**Conclusion:**

Measurement of circulating ActA levels may help identify advanced-stage LUSC patients, and this value could serve as a prognostic parameter in LUSC. Thus, ActA may be a novel blood-based biomarker for identifying LUSC patients with distant metastasis.

## Highlights

• Squamous cell lung cancer management suffers from a lack of biomarkers and molecular targets.
• Plasma levels of activin A are increased in squamous cell lung cancer patients and linked to advanced stage and metastasis.
• Patients with high activin A levels have shorter OS, DFS and PFS.
• High activin A is an independent negative prognostic marker when used alone or when combined in a score with elevated CRP.

## Graphical abstract

**Figure:**
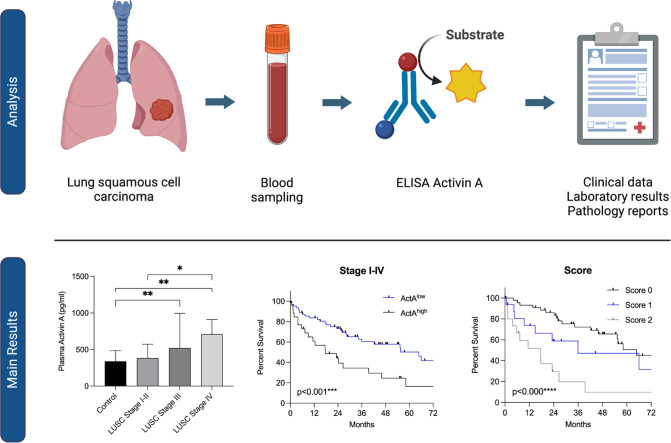

## Introduction

Lung squamous cell carcinoma (LUSC) is the second most common subtype of non-small cell lung cancer (NSCLC) and is still associated with poor prognosis and limited treatment options [1,2]. Although the development of new diagnostic and therapeutic strategies has improved NSCLC treatment and led to longer survival rates, most treatable genetic mutations, such as those in the epidermal growth factor receptor (EGFR) family, anaplastic lymphoma kinase (ALK), and ROS Proto-oncogene 1 (ROS1), are more common in lung adenocarcinoma (LUAD) and are rarely observed in LUSC [3]. Therefore, further investigation into the pathogenesis of LUSC and the identification of novel biomarkers and molecular targets are urgently required.

Activin A (ActA), a member of the transforming growth factor-ß family (TGF-ß), is involved in a broad range of functions including cellular differentiation, homeostasis, and tissue architecture in multiple organs [4]. Furthermore, ActA dysregulation has been reported in various malignancies, although its role in cancer development and progression is controversial and depends on the tumour entity [[5], [6], [7]]. On the one hand, ActA has tumour suppressive effects in breast-, liver- and colon cancer, as well as in diffuse-large cell B-cell lymphoma [5,7,8]. On the other hand, high ActA expression correlates with increased cell growth and proliferation in LUAD, pleural mesothelioma (PM), oesophageal carcinoma, and cancers of the skin, pancreas, ovary, and head and neck [4,[9], [10], [11], [12], [13], [14], [15]]. While it has been previously demonstrated that high circulating ActA levels are a negative prognostic factor in LUAD, PM, and small cell lung cancer (SCLC) [12,16,17], the role of ActA in LUSC has remained largely unknown.

Therefore, in the current study, we investigated circulating ActA levels of LUSC patients and non-malignant controls and demonstrated its usefulness as an independent negative prognostic biomarker in this disease.

## Material and methods

### Patients

We included patients with histologically or cytologically confirmed LUSC (*n* = 128) treated at the Department of Thoracic Surgery, Medical University of Vienna (*n* = 109) and at the National Koranyi Institute of Pulmonology, Budapest (*n* = 19) between 2010 and 2022. Individuals without a history of malignancy (*n* = 73) served as controls. Patients at all disease stages were included in the study. Patients with autoimmune disease, post-stenotic pneumonia, a history of another malignancy within the last 5 years or a history of organ transplantation were excluded. Blood was prospectively collected upon admission for surgical or diagnostic interventions. Clinical data and values for CRP, albumin, and fibrinogen were retrospectively collected from medical and death records provided by the national population-based registry. The term ‘sex’ refers to biological differences, and a binary categorisation (female/male) was used [18]. All the patients were (re)staged according to the 8th edition of the TNM lung cancer classification [19]. Informed consent was obtained from all the patients. This prospective-retrospective study is reported following the REMARK criteria for tumour marker prognostic studies [20,21] and was approved by the ethics committees of the Medical University of Vienna (2009, EK Nr 904/2009) and the National Koranyi Institute of Pulmonology (2018, ETT-TUKEB 23636–2/2018 and 23 636/10/2018/EÜIG) according to the declaration of Helsinki. All the patients provided written informed consent before inclusion in the study. Clinical follow-up was closed on 28 February 2023. The median follow-up was 24.88 months (range 0.13–133.23 months).

### Blood collection

Blood samples were collected through venous puncture into 10 mL ethylenediaminetetraacetic acid (EDTA) Vacutainers (366,448 - BD P100, BD Biosciences, Franklin Lakes, NJ, USA). Within 30 min of blood collection, whole blood samples were centrifuged at 400 g for 15 min at 4 °C. Plasma samples were then centrifuged again at 500 g for 15 min at 4 °C and stored in aliquots at −20 °C until analysis.

### ELISA assays

Quantikine Activin A ELISA kits (DAC00B) were purchased from R&D Systems (Minneapolis, MN, USA). Sample preparation, standard curve generation, and measurement of samples in duplicates were performed in accordance with the manufacturer's recommendations. The performance of the ELISA was consistent with that provided by the manufacturer (https://resources.rndsystems.com/pdfs/datasheets/dac00b.pdf?v=20240207&_ga=2.265486023.999940652.1707388196-212778224.1707388196, accessed 04.06.2024). All the plasma samples yielded results above the sensitivity of the assay (7.85 pg/mL). All measurements were performed in technical duplicate, and a 1:2 dilution was used for all samples.

### Inflammatory markers

The cutoff values for CRP (10 mg/L) and serum albumin (35 g/L) were based on a study by Proctor et al. [22]. The modified Glasgow prognostic score (mGPS) values were defined as follows: mGPS 0, CRP ≤ 10 mg/L and any albumin level; mGPS 1, CRP > 10 mg/L and albumin ≥ 35 g/L; mGPS 2, CRP > 10 mg/L and albumin < 35.

### Activin A gene expression and survival data in the TCGA database

Survival data and activin A gene expression data were extracted from the TCGA database using the UALCAN portal (https://ualcan.path.uab.edu/cgi-bin/TCGA-survival1.pl?genenam=INHBA&ctype=LUSC, downloaded 03.09.2024) [23].

### Statistical analysis

Statistical analyses were performed using the SPSS software (version 27.0, IBM SPSS, IBM Corp., Armonk, NY, USA) and GraphPad Prism (version 9.3.1, GraphPad Prism, GraphPad Software, La Jolla, CA, USA). Normally distributed data are presented as mean ± standard deviation (SD) and non-normally distributed data as median (range). Statistical differences between two independent groups were calculated through the Mann–Whitney U test. The chi-squared test was employed to test for differences between two categorical variables. The Kruskal–Wallis test was used with the post-hoc Dunn test to compare more than two groups. The Bonferroni correction was utilised to adjust for type I alpha errors. We defined overall survival (OS) as the time from surgery for patients undergoing curative intent resection or from diagnostic intervention for patients with advanced stages to the date of death from any cause. Patients who were still alive were censored at the time of their last contact. Disease-free survival (DFS) was calculated from the time of surgery to the time of pathologically or clinically proven recurrence for patients undergoing curative-intent surgery, and progression-free survival (PFS) was calculated from the time of diagnosis until progression for patients with advanced stages treated through systemic therapy. OS, DFS, and PFS were analysed using the Kaplan–Meier method, and differences between groups were compared with the log-rank test. Univariate and multivariate Cox regression were used to calculate hazard ratios (HRs) and 95 % confidence intervals (CI) for survival analysis and to explore prognostic clinical factors. The optimal cutoff values of ActA for OS and DFS/PFS were estimated using the X-tile software (Yale University, USA) according to the minimal p-value approach [24]. Receiver-operating characteristic (ROC) curve analysis was performed to assess the diagnostic relevance of ActA. Statistical significance was defined as a two-tailed p-value of <0.05. Graphical illustrations were created using GraphPad Prism software.

## Results

### Patient characteristics

**Fig. 1:**
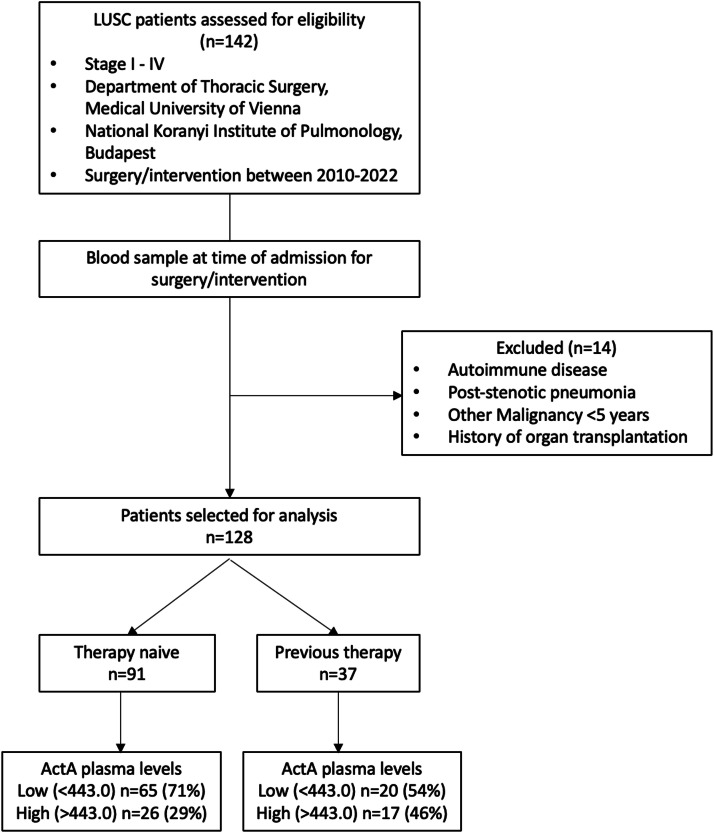
Consort diagram to demonstrate patient selection LUSC, lung squamous cell carcinoma; ActA, activin A.

Between 2010 and 2022, 142 LUSC patients were identified at both participating centres. Fourteen patients were excluded on the basis of the exclusion criteria (Fig. 1). In the remaining cohort (*n* = 128), the majority of patients were male (*n* = 95, 74.2 %), with a median age of 66.19 at time of surgery or intervention. Moreover, 91 (71.1 %) patients were therapy naive at time of blood withdrawal, whereas 37 (28.9 %) patients had undergone previous therapy. A CONSORT diagram is shown in Fig. 1. Most patients underwent curative-intent surgical resection (*n* = 111, 86.7 %). Patient characteristics are listed in Table 1 for treatment naive patients and in Supplementary Table 1 for patients who had received previous therapy.

**Table 1:** Clinicopathological characteristics of therapy naive LUSC patients by plasma ActA levels.

	ActA^low^ (*n* = 65)		ActA^high^ (*n* = 26)			All patients (*n* = 91)	
	n	%	n	%	P	n	%
Gender Male Female	50 15	77 % 23 %	16 10	62 % 38 %	0.193	66 25	72.5 % 27.5 %
Age <65 ≥65	27 38	42 % 58 %	10 16	38 % 62 %	0.745	37 54	41 % 59 %
Smoking status Current Former Never	29 33 3	45 % 51 % 4 %	17 8 1	65 % 31 % 4 %	0.196	46 41 4	51 % 45 % 4 %
Stage I II III IV	21 25 18 1	32 % 38 % 28 % 2 %	4 9 8 5	15 % 35 % 31 % 19 %	**0.012**	25 34 26 6	27 % 37 % 29 % 7 %
Treatment Surgery Systemic therapy	58 7	89 % 11 %	17 9	65 % 35 %	**0.007**	75 16	82 % 18 %

LUSC, lung squamous cell carcinoma.

ActA, Activin A.

### ActA levels are elevated in LUSC patients

**Fig. 2:**
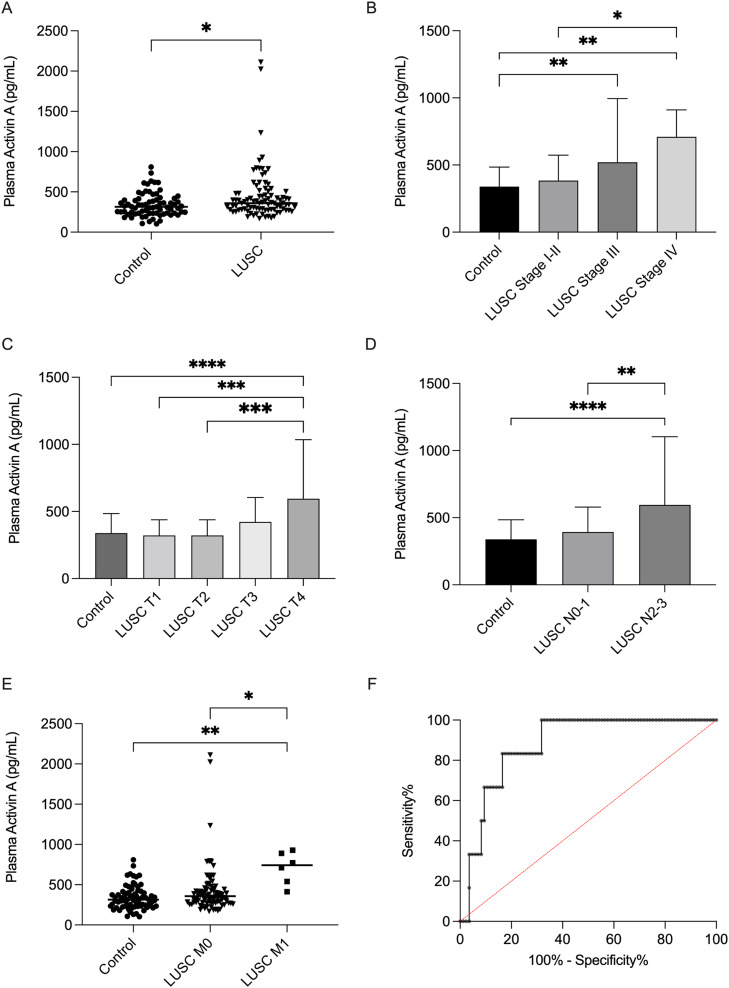
ActA plasma concentration in therapy naive LUSC patients. A) ActA plasma concentration was significantly higher in LUSC patients than in the control group (*p* = 0.010). B), C), D) Stage- as well as T- and N-status-dependent increase of plasma ActA levels in LUSC patients. E) ActA plasma concentration was elevated in therapy naive LUSC patients with M1 disease compared to that in controls (*p* = 0.002) or M0 patients (*p* = 0.019). F) Circulating ActA levels may be a useful biomarker to identify LUSC patients with distant metastasis compared to non-metastatic LUSC patients, AUC 0.878 (95 % CI 0.784–0.973, *p* = 0.002). *p < 0.05, **p < 0.01, ***p < 0.001, ****p < 0.0001; ActA, activin A; LUSC, lung squamous cell carcinoma.

To analyse a more homogeneous study cohort, we first focused on therapy naive LUSC patients. The ActA levels of 91 LUSC patients were measured and compared with those in 73 individuals without malignant disease. The mean ActA concentration was significantly higher in LUSC patients (444.1 ± 310.9 pg/mL; range: 168.3–2111.0 pg/mL) than in the control group (338.9 ± 145.5 pg/mL; range: 103.8–808.8 pg/mL; *p* = 0.010, Fig. 2A). The clinicopathological characteristics of therapy naive LUSC patients according to dichotomised ActA subgroups are presented in Table 1. Patients were divided into subgroups with high and low ActA levels based on the calculation of the optimal cutoff value using X-tile software. This calculation identified 443.0 pg/mL as the optimal cutoff value. No statistically significant associations were detected between sex, age, or smoking status. However, plasma ActA levels were significantly correlated with tumour stage. We analysed the impact of TNM classification on ActA concentration and detected a stage- and T and N status-dependent increase in circulating ActA levels (Fig. 2B–D). ActA levels were significantly elevated in stage III and IV patients compared to those in the control group (*p* = 0.007 and *p* = 0.002, respectively), as well as in stage IV patients compared to those in the early stages (stage I-II, *p* = 0.011) (Fig. 2B). ActA plasma levels were also significantly increased in T4 LUSC patients compared to controls or T1 and T2 disease patients (Fig. 2C). Furthermore, LUSC patients with positive N2-N3 lymph nodes showed significantly higher ActA concentrations than those with N0-N1 lymph nodes or controls (*p* = 0.002 and *p* < 0.0001, Fig. 2D). Importantly, ActA plasma levels were significantly higher in LUSC patients with distant metastases than in controls (*p* = 0.002) or M0 patients (*p* = 0.019, Fig. 2E). ROC curve analysis was performed to evaluate the diagnostic accuracy of circulating ActA in metastatic diseases. It showed a sensitivity of 83.33 % (95 % CI 43.65–99.15 %) and a specificity of 74.12 % (95 % CI 63.91–82.24 %) for the cutoff value of 443.0 pg/mL to distinguish patients with distant metastasis from M0 patients. The area under the curve (AUC) value was 0.878 (95 % CI 0.784–0.973, *p* = 0.002) (Fig. 2F).

Next, we extended our analysis to plasma ActA levels in LUSC patients who had received anticancer treatment before blood withdrawal (*n* = 37) compared to those in the control group (*n* = 73). The mean plasma concentration was significantly higher in LUSC patients compared to that in the controls (506.8 ± 320.2 pg/mL; range: 125.1–1631.0 pg/mL vs 338.9 ± 145.5; range: 103.8–808.8; *p* = 0.0007, Supplementary Figure 1A). The characteristics of LUSC patients after previous therapy are presented in Supplementary Table 1. Firstly, we analysed the correlation between circulating ActA levels and TNM classification and detected a stage- as well as T-dependent increase in circulating ActA levels (Supplementary Figure 1B-C). Plasma ActA levels were significantly elevated in stage I-II and III-IV patients compared to that in the control group (*p* = 0.013 and 0.046, Supplementary Figure 1B). However, there was no significant difference between LUSC patients with pathological complete response after previous therapy (pCR, ypT0 ypN0 M0, stage 0) compared to that in controls or other stage patients (Supplementary Figure 1B). Furthermore, in LUSC patients after previous therapy, ActA plasma levels were also significantly increased at T0-T2 and T3-T4 compared with those in controls (*p* = 0.026 and 0.004, Supplementary Figure 1C). Additionally, the patients were further stratified by the cutoff value of 443.00 into ActA^high^ and ActA^low^ subgroup as described in Section 3.1. There were no significant correlations between the ActA^high^ and ActA^low^ subgroups with tumour stage, treatment, sex, age, or smoking status (Supplementary Table 1).

### High circulating ActA A levels are independently associated with poor survival in therapy naive LUSC patients

**Fig. 3:**
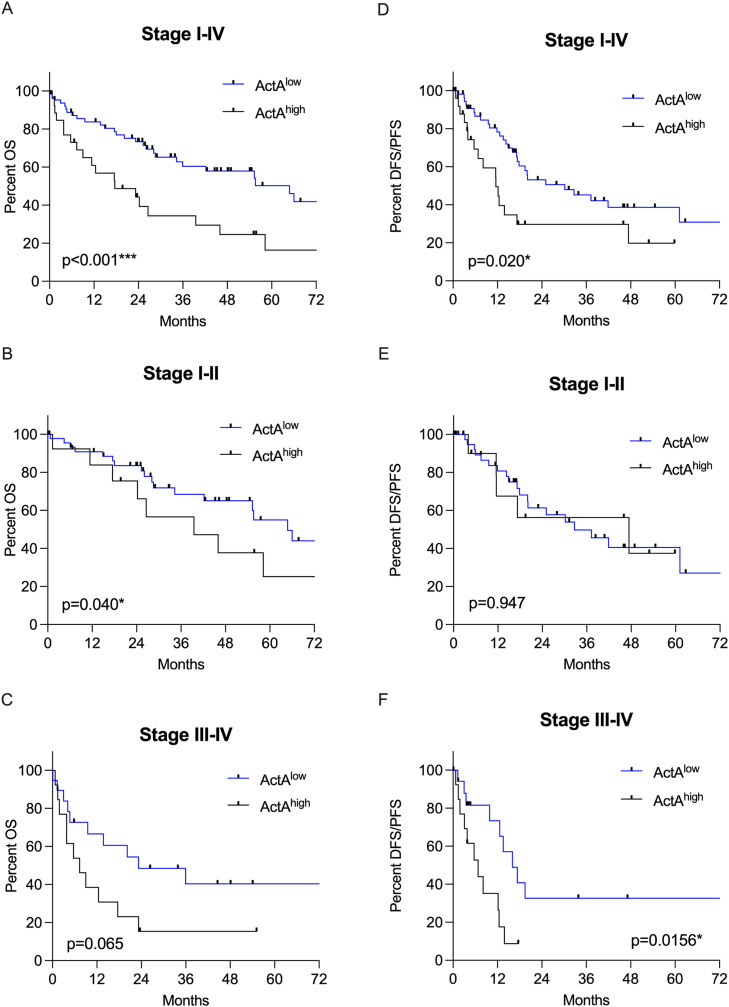
Kaplan–Meier curves for OS and DFS/PFS of therapy naive LUSC patients according to plasma ActA levels (cut-off value 443.0). A) LUSC patients with high (>443.0 pg/mL) plasma ActA levels had significantly shorter OS than those with low plasma ActA levels (17.63 vs 64.77 months, HR 0.391, 95 % CI 0.200–0.762, *p* < 0.001). B) Early stage LUSC patients with elevated plasma ActA levels had significantly worse OS (39.50 vs 64.77, HR 0.465, 95 % CI 0.186–1.160, *p* = 0.040). C) A trend for longer OS in the plasma ActA^low^ subgroup in LUSC patients with stage III-IV was observed (23.23 vs 7.33 months, HR 0.462, 95 % CI 0.186–1.141, *p* = 0.065). D) LUSC patients with high (>443.0 pg/mL) plasma ActA levels had significantly shorter DFS/PFS than those with low plasma ActA levels (median DFS/PFS 11.57 vs 30.20 months, HR 0.502, 95 % CI 0.248–1.019, *p* = 0.020). E) In early stage LUSC patients with elevated plasma ActA levels, there was no significant difference regarding DFS/PFS (median DFS/PFS 47.4 vs 32.67 months, HR 0.968, 95 % CI 0.340–2.604, *p* = 0.947). F) LUSC patients with stage III-IV in the plasma ActA^low^ subgroup had a significantly longer DFS/PFS (median DFS/PFS 6.77 vs 16.03 months, HR 0.391, 95 % CI 0.154–0.991, *p* = 0.0156. ActA, activin A; OS, overall survival; DFS, disease free survival; PFS, progression free survival; CI, confidence interval; HR, hazard ratio; LUSC, lung squamous cell carcinoma.

Next, we investigated the impact of high circulating ActA levels on OS in all therapy naive LUSC patients. When patients were categorised into an ActA^low^ and ActA^high^ subgroups (cutoff 443.0 pg/mL), patients with high ActA plasma levels had significantly shorter OS compared to those with low concentrations (median OS 17.63 vs 64.77 months, HR: 0.391, 95 % CI 0.200–0.762, *p* < 0.001, Fig. 3A). These results are in line with the gene expression and survival data from the TCGA database analysed on the ULCAN portal (Supplementary Figure 2). Stage-dependent subgroup analysis revealed that stage I-II LUSC patients with elevated ActA plasma concentrations continued to have a significantly decreased OS (median OS 39.50 vs 64.77 months, HR 0.465, 95 % CI 0.186–1.160, *p* = 0.040, Fig. 3B), while in advanced stages, a trend towards shorter OS for patients with high ActA plasma levels did not reach statistical significance (median OS 7.33 vs 23.23 months, HR 0.462, 95 % CI 0.186–1.141, *p* = 0.065, Fig. 3C).

Additionally, we performed Kaplan–Meier analysis for DFS/PFS, which revealed that patients with high ActA levels had significantly shorter DFS/PFS than those with low ActA levels (median DFS/PFS 11.57 vs 30.20 months, HR 0.502, 95 % CI 0.248–1.019, *p* = 0.020, Fig. 3D). Furthermore, subgroup analysis showed that in early stage LUSC patients, no significant difference regarding median DFS/PFS (47.4 vs 32.67 months, HR 0.968, 95 % CI 0.340–2.604, *p* = 0.947, Fig. 3E) between subgroups was noted, whereas in advanced stages (stage III-IV), the plasma ActA^high^ subgroup had a significantly shorter median DFS/PFS (6.77 vs 16.03 months, HR 0.391, 95 % CI 0.154–0.991, *p* = 0.0156, Fig. 3F).

As an additional subgroup analysis, we investigated only patients who were treatment-naïve and underwent curative-intent surgery (stages I-III). Patients with high ActA levels had significantly shorter OS compared to those with low ActA levels (26.57 vs 64.77 months, HR 0.340, 95 % CI 0.148–0.780, *p* = 0.011, Supplementary Figure 3A); however there was no significant difference in DFS (37.27 vs 47.40 months, HR 0.786, 95 % CI 0.340–1.818, *p* = 0.513, Supplementary Figure 3B).

Multivariable analysis, including age, sex, tumour stage, and plasma ActA concentrations, revealed that high ActA plasma levels were independent prognostic factors for OS (*p* = 0.001) and DFS/PFS (*p* = 0.018). Moreover, tumour stage was an independent prognostic factor for OS (*p* = 0.002) and DFS/PFS (*p* < 0.0001), and sex was an independent prognostic factor for DFS/PFS (*p* = 0.027), as shown in Table 2.

**Table 2:** Multivariate Cox regression survival analysis for patient characteristics of therapy naive LUSC patients (*n* = 91).

	HR	95 % CI			P
		Lower	Upper		
Overall survival					
Age (continuous)	0.987	0.955		1.020	0.438
Gender	1.629	0.828		3.205	0.157
Stage I-II vs III-IV	0.363	0.193		0.681	**0.002**
ActA levels ActA^low^ vs ActA^high^	0.380	0.213		0.678	**0.001**
**Disease-/Progression-free survival**					
Age (continuous)	0.987	0.950		1.025	0.491
Gender	2.269	1.097		4.692	**0.027**
Stage I-II vs III-IV	0.301	0.159		0.570	**<0.001**
ActA levels ActA^low^ vs ActA^high^	0.465	0.246		0.878	**0.018**

HR, hazard ratio; CI, confidence interval; LUSC, lung squamous cell carcinoma; ActA, Activin A.

We also performed a survival analysis in LUSC patients after previous therapy based on the ActA^high^ and ActA^low^ subgroups, wherein we could not detect statistical differences in OS or PFS between these groups. However, a trend toward better OS was observed in the ActA^low^ cohort than in the ActA^high^ cohort (stage 0-IV; stage 0=pCR) (*p* = 0.0803, Supplementary Figure 4).

### Correlation of ActA A concentration with inflammatory markers

**Fig. 4:**
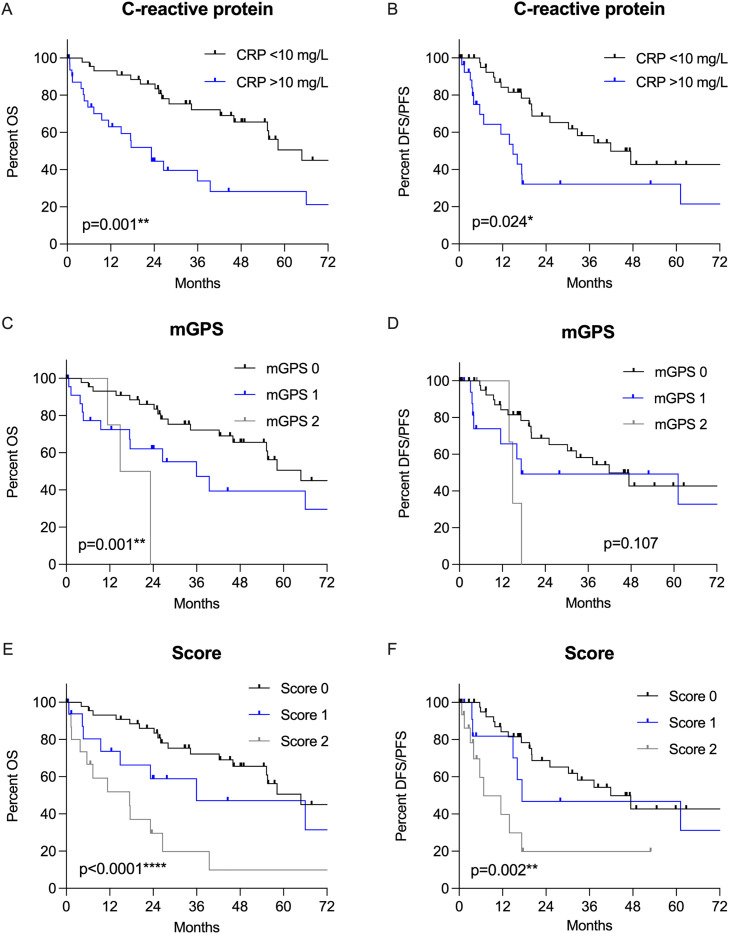
Kaplan–Meier curves of therapy naive LUSC patients for A) OS (*p* = 0.001) and B) DFS/PFS (*p* = 0.024) according to elevated CRP levels, C) OS (*p* = 0.001) and D) DFS/PFS (*p* = 0.107) according to the mGPS, and E) OS (*p* < 0.0001) and F) DFS/PFS (*p* = 0.002) according to our newly developed score. Score 0, CRP ≤ 10 mg/L and any ActA level; score 1, CRP > 10 mg/L and ActA^low^; score 2, CRP > 10 mg/L and ActA^high^. ActA, activin A; OS, overall survival; DFS, disease free survival; PFS, progression free survival; LUSC, lung squamous cell carcinoma; mGPS, modified Glasgow prognostic score.

In 41.6 % of therapy naive LUSC patients, CRP was elevated (CRP >10 mg/L, *n* = 32/77, data missing in 14 patients), which was significantly associated with high ActA levels (*p* = 0.001) and with decreased OS and DFS/PFS (*p* = 0.001 and *p* = 0.024, Fig. 4A–B). We further analysed possible correlations with the well-established mGPS. Twenty-seven patients had an elevated mGPS (mGPS 1–2, 37.5 %, *n* = 27/72, data missing for 19 patients), which showed a significant correlation with the ActA^high^ subgroup (mGPS 1–2 vs mGPS 0, *p* = 0.008) and was linked to significantly shorter OS (*p* = 0.001) but not with DFS/PFS (Fig. 4C–D). Conversely, elevated fibrinogen levels (>40 mg/L, 72.2 %, *n* = 52/72, data missing for 19 patients) showed no correlation with high ActA levels (*p* = 0.429).

### New prognostic score based on C-reactive protein and ActA levels is independently associated with survival in therapy naive LUSC patients

Based on the significant results regarding elevated CRP and high ActA levels, a new prognostic score was devised based on the combination of CRP and ActA levels as follows: score 0, CRP ≤ 10 mg/L and any ActA level; score 1, CRP > 10 mg/L and ActA^low^; and score 2, CRP > 10 mg/L and ActA^high^. In our study cohort of therapy naive LUSC patients, the majority of patients (58.4 %, *n* = 45/77) had a score of 0; 17 patients (22.1 %), score of 1; and five patients, score of 2 (CRP values were missing in 14 patients). The median OS was 64.8 months (95 % CI 48.5–81.1) for patients with score 0, 36.0 months (95 % CI 0.0–81.1) with score 1, and 17.5 months (95 % CI 0.0–35.2) with score 2. The differences in survival probabilities between scores of 0, 1, and 2 were statistically significant (*p* < 0.0001; Fig. 4E). There was also a statistically significant difference between the DFS/PFS based on the score (*p* = 0.002) with a median DFS/PFS of 41.9 months (95 % CI 24.6–59.2) for patients with score 0, 17.4 months (95 % CI 0.0–71.5) with score 1, and 6.8 months (95 % CI 0.0–15.4) with score 2 (*p* = 0.002, Fig. 4F). Comparing the survival differences regarding elevated CRP, mGPS, ActA alone, and our new score, the score showed better differentiation between the groups for OS as well as for DFS/PFS (Fig. 4).

In multivariate analysis (score, age, sex, and tumour stage), our score (HR 1.9, 95 % CI 1.27–2.89, *p* = 0.002), tumour stage (HR 1.8, 95 % CI 1.1–2.9, *p* = 0.021), and sex (HR 2.2, 95 % CI 1.0–4.7, *p* = 0.045) were independently associated with OS. Regarding DFS/PFS, our score (HR 1.7, 95 % CI 1.1–2.8, *p* = 0.016) and sex (HR 2.3, 95 % CI 1.0–5.4, *p* = 0.049) were independently significant, whereas tumour stage showed only a trend (HR 1.6, 95 % CI 0.97–2.6, *p* = 0.065).

## Discussion

To date, systemic therapeutic options for LUSC remain scarce, because there are no targeted therapeutic agents for the specific treatment of LUSC. Additionally, the availability of diagnostic and prognostic blood biomarkers is limited. Blood biomarkers currently used for LUSC include carcinoembryonic antigen, cytokeratin 19 fragment, squamous cell carcinoma antigen, and neuron-specific enolase; however, despite extensive research efforts, there are still no valid biomarkers for clinical patient management [2,[25], [26], [27], [28], [29], [30]].

ActA is an intriguing molecule implicated in a plethora of functions in cancer cells and as a regulator of interactions in the tumour microenvironment. Although both pro- and anti-tumourigenic functions of ActA have been reported, malignant tumours often show ActA upregulation, which correlates with a worse prognosis [5,31]. In contrast, in diffuse large B-cell lymphoma, reduced ActA expression was found to correlate with reduced OS and PFS [32] and several malignancies, including breast and hepatocellular carcinoma, express high levels of ActA antagonists, such as follistatin and follistatin-like 3 [33,34].

Our group has previously investigated ActA in the plasma and/or serum of patients with LUAD, SCLC, and PM, with similar results for plasma and serum [12,16,17]. In this study, plasma was used because of its superior availability. In LUAD, SCLC, and PM, ActA was upregulated and linked to more advanced disease and worse prognosis. The reported plasma values in the patient cohorts of these studies were 562 pg/mL (mean, LUAD), 562 pg/mL (median, PM), and 548 pg/mL (median, SCLC), which were higher than those in the current study, whereas the values in the controls were essentially similar. Owing to the correlation between ActA and disease stage, the number of early-stage versus late-stage patients in different cohorts will influence the results.

Despite the frequent elevation of ActA in the plasma of cancer patients, the contribution of cancer cells versus various stromal compartments to elevated ActA levels remains unclear. Elevated ActA expression has been reported in cancer cells of various malignancies. Cancer-associated fibroblasts, endothelial cells, macrophages, and other immune cells have been described to express ActA and could contribute to elevated ActA plasma levels [6,35,36]. Owing to its multiple pro-tumourigenic functions, high ActA levels could causally contribute to cancer progression by promoting growth, migration, and invasion, inducing stemness and drug resistance, or contributing to immune evasion [5]. There has been only little research published on functional aspects of ActA in LUSC. Taniguchi et al. reported that the upregulation of ActA in lung alveolar macrophages increases the proliferation of lung cancer cells in vivo and similar results were also shown in an orthotopic model with a specific squamous cell carcinoma cell line [37]. Moreover, similar tumour promoting results are published in other types of thoracic cancer including PM and LUAD [16,38,39].

ActA has been linked to both acute and chronic inflammatory conditions, including sepsis [40], asthma [41], and COPD [42,43] providing a strong rationale for its investigation in LUSC, as inflammation is considered a key element in the pathogenesis of this disease [[44], [45], [46]]. Interestingly, no correlation between ActA and fibrinogen, which has been previously reported in PM [12], was found in this study. Another inflammation-related biomarker that has been investigated in NSCLC, especially in LUSC, is CRP [[47], [48], [49], [50], [51]]. Additionally, the combination of elevated CRP levels and a decrease in albumin, known as mGPS, has been reported to have prognostic value in several malignancies, including NSCLC [[52], [53], [54], [55]]. First, we analysed these inflammatory markers in our cohort, which showed significant results for elevated CRP levels regarding OS and DFS/PFS (*p* = 0.001 and *p* = 0.024) and significant differences between the mGPS score values for OS (*p* = 0.001) but not for DFS/PFS (*p* = 0.107). Therefore, we investigated the prognostic performance of the combination of elevated CRP and ActA levels to establish a more specific score, with the intention of identifying patients with worse prognosis. Indeed, the addition of ActA to CRP led to a more accurate differentiation of patient prognosis compared to CRP alone or mGPS. This proposed score may help identify patients at a higher risk of recurrence and worse survival with the need for more aggressive therapeutic approaches or closer follow-up times.

In our multivariate analysis sex and age were also used as covariables as several studies have demonstrated their influence on survival in NSCLC patients. It has been repeatedly reported that women have better survival rates compared to men [[56], [57], [58], [59], [60]]. Our results confirmed the female sex as an independent prognostic factor for DFS/PFS, but not for OS in therapy naïve LUSC patients analysing the effect of high ActA levels and for both DFS/PFS and OS analysing our new prognostic score. However, we could not identify a significant correlation between sex and high ActA levels alone, which is in line with findings of another study focusing on sex differences and ActA levels in pancreatic cancer [61]. Age could not be identified as an independent prognostic factor in our analysis. This could be explained by a wide age range (25–87 years) and a selection bias of fitter patients due to the surgical department as performing centre of this study. This is also in line with the results of a previous study [62].

This study had several limitations. Firstly, this was a prospective retrospective study; blood was sampled prospectively, but clinical data were collected retrospectively. Secondly, the study was mainly performed at the Department of Thoracic Surgery, and most patients were referred for surgery or intervention from different centres. Therefore, the majority of patients had stage I-II disease, resulting in a cohort with a limited number of advanced stage patients (especially stage IV disease, *n* = 7). Moreover, blood samples were collected after previous therapy upon admission to our institution. Lastly, we did not have blood samples from different time points (for example, at the time of diagnosis, after therapy, and at the time of progression); therefore, it remains unclear whether ActA could serve as a potential biomarker for monitoring the disease course. Nevertheless, the obtained data clearly suggest that ActA is elevated in LUSC patients and correlates with a more advanced stage as well as with T, N, and M factors. Moreover, patients with high ActA levels exhibited poorer survival rates.

## Conclusion

Elevated plasma ActA levels significantly correlated with tumour stage in treatment naive LUSC patients and were independent prognostic factors for shorter OS and DFS/PFS. Measurement of circulating ActA plasma levels may help to identify LUSC patients with advanced disease stages and determine their prognosis. Consequently, ActA levels may serve as a novel biomarker for identifying LUSC patients with distant metastasis and a promising non-invasive tool for monitoring patients. Combining ActA levels with other inflammatory biomarkers appears promising and should be explored further.

## Funding

This work was supported by the “Fonds der Stadt Wien für Innovative Interdisziplinäre Krebsforschung” (Nr 21199) to Katharina Sinn and by the Hungarian National Research, Development and Innovation Office (KH130356, KKP126790, 2020-1.1.6-JÖVŐ and TKP2021-EGA-33) and by the 10.13039/501100002428Austrian Science Fund (FWF I3522, FWF I3977 and I4677) to Balazs Dome.

## CRediT authorship contribution statement

**Katharina Sinn:** Writing – review & editing, Writing – original draft, Visualization, Project administration, Investigation, Funding acquisition, Data curation, Conceptualization. **Ahmed Elbeialy:** Writing – review & editing, Validation, Investigation. **Berta Mosleh:** Writing – review & editing, Validation, Investigation. **Clemens Aigner:** Writing – review & editing, Supervision, Resources. **Karin Schelch:** Writing – review & editing, Validation, Investigation. **Viktoria Laszlo:** Writing – review & editing, Validation, Investigation. **Balazs Dome:** Writing – review & editing, Supervision, Project administration, Conceptualization. **Mir Alireza Hoda:** Writing – review & editing, Validation, Supervision, Project administration, Data curation, Conceptualization. **Michael Grusch:** Writing – review & editing, Writing – original draft, Supervision, Project administration, Funding acquisition, Conceptualization.

## Declaration of competing interest

All authors declare that they have no competing interests.

## Appendix Supplementary materials

Image, application 1
