# Supplementary material for: High circulating activin A plasma levels are associated with tumour stage and poor survival in treatment-naive lung squamous cell cancer patients
Source: Transl Oncol. 2024 Oct 15;51:102153. doi: 10.1016/j.tranon.2024.102153 (PMC11525229; doi:10.1016/j.tranon.2024.102153)
Supplement: Supplementary file 1 [file mmc1.docx]

**Supplementary Table 1:** Clinicopathological characteristics of LUSC patients after previous therapy by plasma ActA levels

|  | ActA^low^  (n=20) | | ActA^high^  (n=17) | |  | All patients  (n=37) | |
| --- | --- | --- | --- | --- | --- | --- | --- |
|  | n | % | n | % | p | n | % |
| Gender  Male  Female | 14  6 | 70%  30% | 14  3 | 82%  18% | 0.383 | 28  9 | 76%  24% |
| Age  <65  ≥65 | 13  7 | 65%  35% | 10  7 | 59%  41% | 0.699 | 23  14 | 62%  38% |
| Smoking status  Current  Former  Never  Unknown | 7  12  1  0 | 35%  60%  5%  0% | 6  8  2  1 | 35%  47%  12%  6% | 0.679 | 13  20  3  1 | 35%  54%  8%  3% |
| Stage  pCR  I  II  III  IV | 2  3  8  7  0 | 10%  15%  40%  35%  0% | 2  3  5  6  1 | 12%  18%  29%  35%  6% | 0.820 | 4  6  13  13  1 | 11%  16%  35%  35%  3% |
| Previous Treatment  Neoadj. CHT  Neoadj. IO  Neoadj. CHT+IO  Neoadj. CHT+RT  Neoadj. CHT+IO+RT Palliative systemic treatment* | 13  0  3  3  1  0 | 65%  0%  15%  15%  5%  0% | 11  1  3  1  0  1 | 64%  6%  18%  6%  0%  6% | 0.763 | 24  1  6  4  1  1 | 65%  3%  16%  10%  3%  3% |

LUSC, lung squamous cell carcinoma; ActA, Activin A; pCR, pathological complete response

neoadj., neoadjuvant; CHT, chemotherapy; IO, immunotherapy; RT, radiotherapy
*Patient underwent bronchoscopic coring out for symptom control in a palliative setting


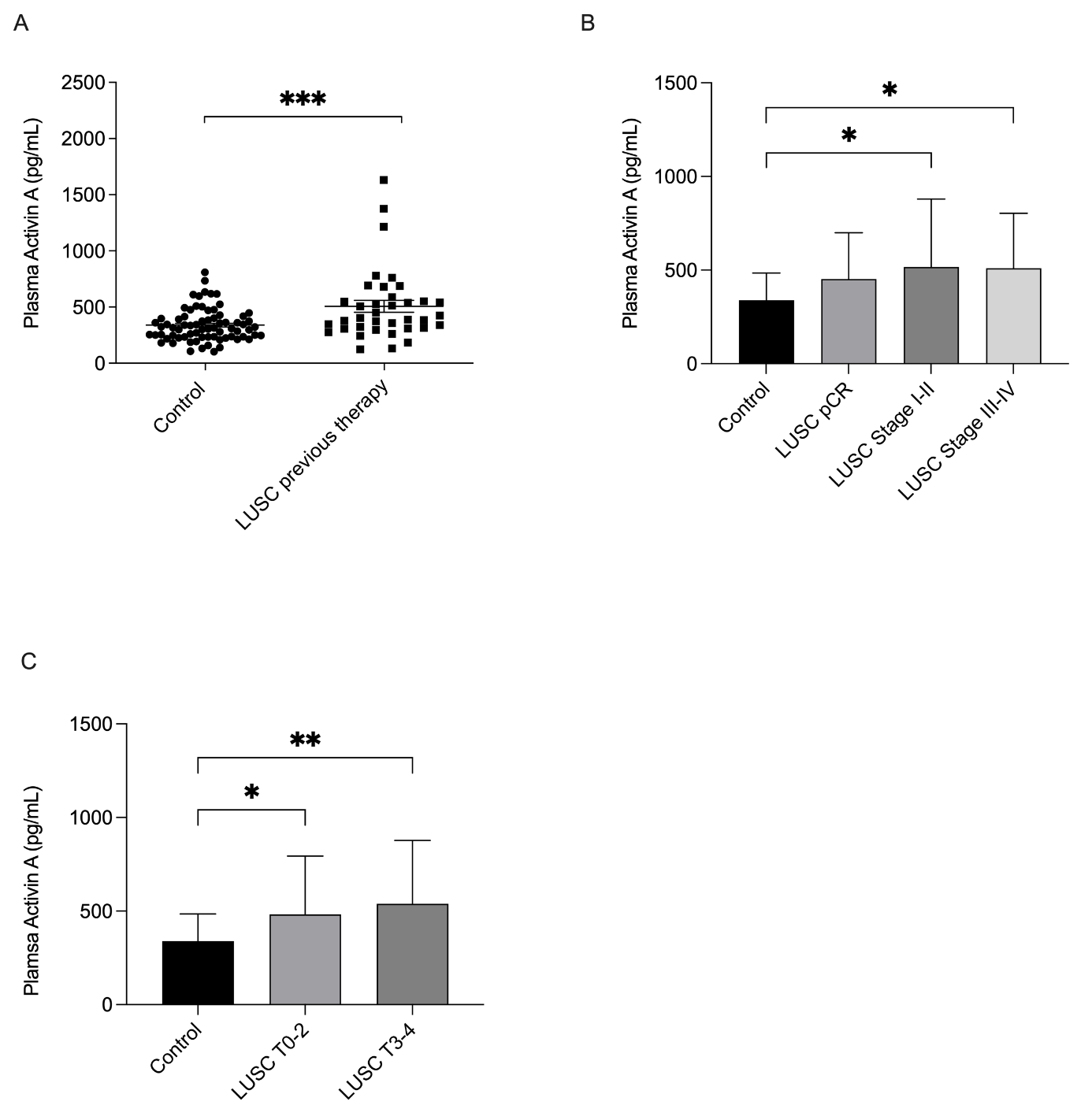


**Supplementary Figure 1:** ActA plasma concentration in LUSC patients after previous therapy. A) ActA plasma concentration is significantly higher in LUSC patients after previous therapy than in the control group (p=0.0007). B) Stage- dependent increase of plasma ActA levels in LUSC patients after previous therapy compared to controls. C) ActA plasma levels were significantly increased in T0-T2 and T3-T4 compared to the control group (p=0.026 and 0.004). *p<0.05, **p<0.01, ***p<0.001

ActA, Activin A; LUSC, lung squamous cell carcinoma; pCR, pathological complete response = ypT0 ypN0 M0.

**Supplementary Figure 2:** Kaplan-Meier survival analysis of LUSC patients according to activin A gene expression levels from the TCGA database. Patients with high expression level had a significant lower survival compared to those with low/medium expression (p=0.01).

Activin A; LUSC, lung squamous cell carcinoma


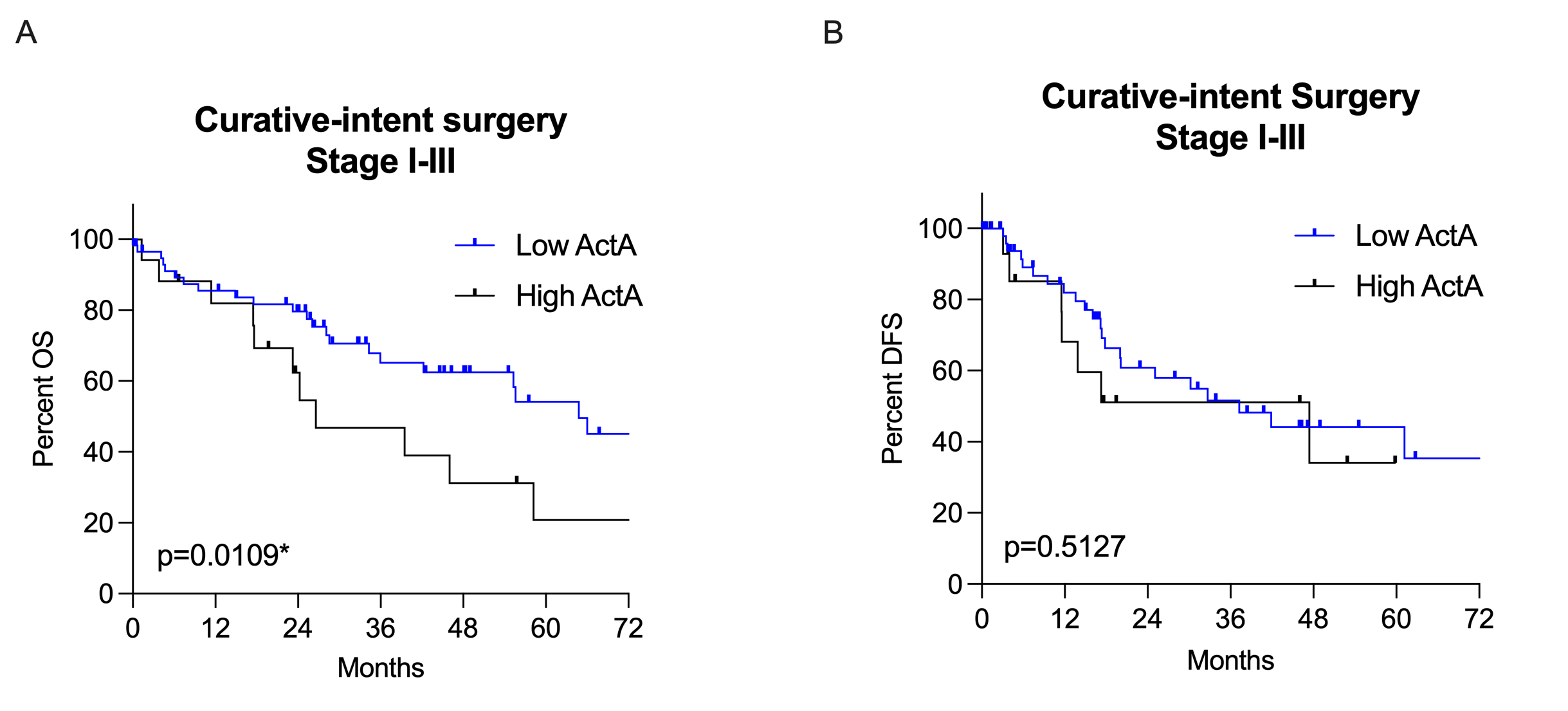


**Supplementary Figure 3:** Kaplan-Meier curves for OS and DFS of therapy naive LUSC patients who underwent curative-intent surgery according to plasma ActA levels (cut-off value 443.0). A) LUSC Patients in the ActA^high^ subgroup had significantly worse OS compared to patients in the ActA^low^ subgroup (26.57 vs 64.77 months, HR 0.340, 95% CI 0.148-0.780, p=0.011). B) No significant difference in DFS between the subgroups was detected (37.27 vs 47.40 months, HR 0.786, 95% CI 0.340-1.818, p=0.513)

OS, Overall survival; DFS, Disease-free survival; ActA, Activin A; LUSC, lung squamous cell carcinoma.


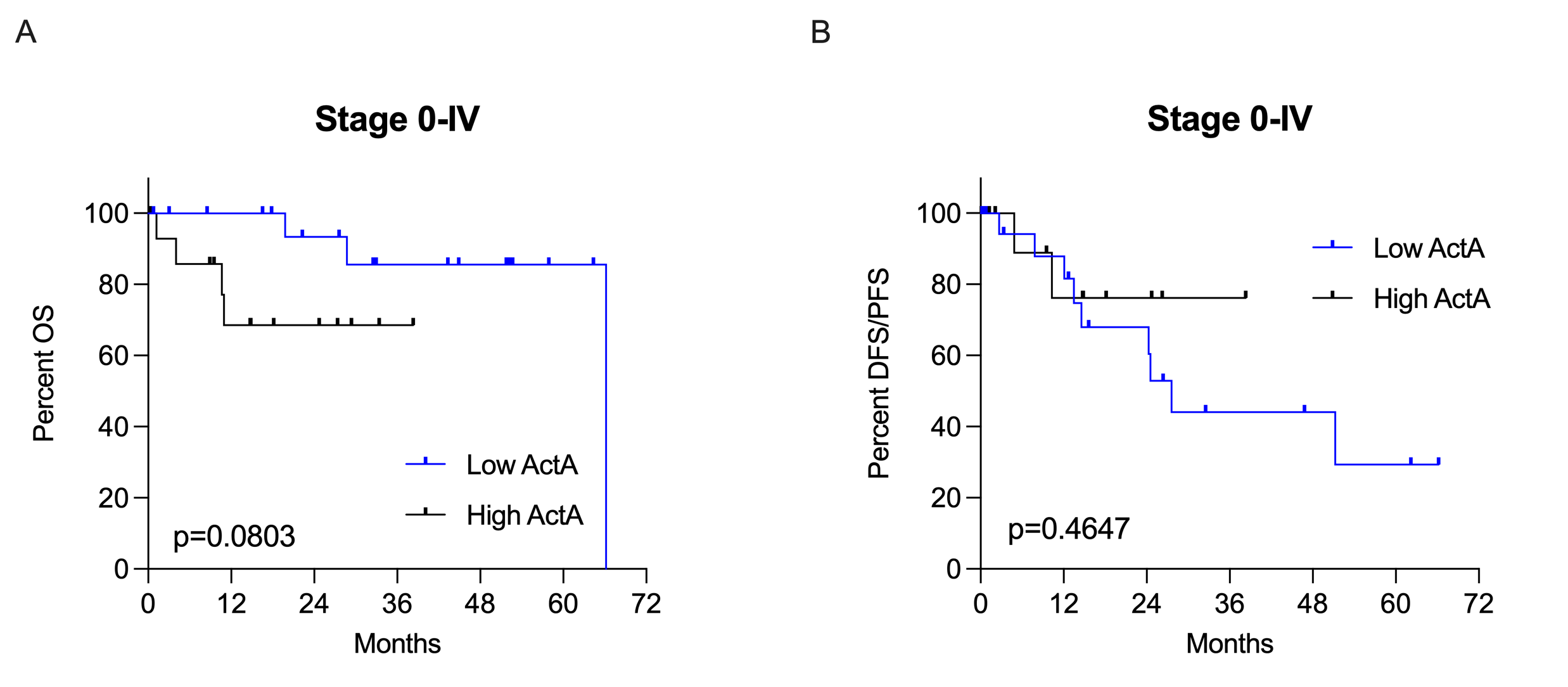


**Supplementary Figure 4:** Kaplan-Meier curves for OS and PFS of LUSC patients after previous therapy according to plasma ActA levels (cut-off value 443.0). A) There is a trend for better OS in the ActA^low^ cohort compared with the ActA^high^ subgroup. B) No difference regarding PFS between both subgroups was detected.

OS, Overall survival; PFS, Progression-free survival; ActA, Activin A; LUSC, lung squamous cell carcinoma; Stage 0 = pathological complete response = ypT0 ypN0 M0
